# Sociodemographic and Temporal Differences in Menthol Cigarette Use Among US Adults Who Smoke, 1999–2018

**DOI:** 10.5888/pcd21.230291

**Published:** 2024-03-28

**Authors:** Yiling J. Cheng, James Tsai, Monica E. Cornelius, Margaret Mahoney, Linda J. Neff

**Affiliations:** 1 Office on Smoking and Health, National Center for Chronic Disease Prevention and Health Promotion, Centers for Disease Control and Prevention, Atlanta, Georgia; 2 Katmai Government Services, Centers for Disease Control and Prevention, Office on Smoking and Health, Atlanta, Georgia

## Abstract

**Introduction:**

Monitoring menthol cigarette use allows for identification of potential health disparities. We examined sociodemographic and temporal differences in menthol cigarette use among US adults who smoke.

**Methods:**

We analyzed data from the 1999–2018 National Health and Nutrition Examination Survey for adults aged 20 years or older who smoke (N = 11,431) using binary logistic regression.

**Results:**

Among US adults who smoke, 28.8% used menthol cigarettes. After adjusting for age, sex, race and ethnicity, education, income-to-poverty ratio, and health status, the prevalence of menthol use among adults who smoke increased on average by 3.8% (95% CI, 2.7%–4.9%) annually. Non-Hispanic Black adults had the highest average prevalence of menthol cigarette use, 73.0% (95% CI, 70.9%–75.2%), and Mexican American adults had higher average annual increase in menthol cigarette use, 7.1% (95% CI, 4.0%–10.3%). Adults with fair or poor health status had a 4.3% annual increase in menthol cigarette use (95% CI, 2.5%–6.1%). The adjusted prevalence ratios of menthol cigarette use were 1.61 (95% CI, 1.39–1.83) for adults aged 20–29 years compared with those aged 65 years or older, 1.41 (95% CI, 1.32–1.49) for female adults compared with male adults, and 1.17 (95% CI, 1.07–1.27) for high school graduates or higher compared with those with no high school diploma.

**Conclusion:**

Non-Hispanic Black adults who smoke had the highest prevalence of menthol cigarette use among all racial and ethnic groups; the prevalence of menthol cigarette use among adults who smoke increased especially among Mexican American adults, younger adults, and adults who reported fair to poor health status.

**Summary**

**What is already known on this topic?**

Menthol cigarette use has increased over the past few decades. The commercial tobacco industry targets menthol tobacco products to specific demographic groups, and disparities exist in menthol cigarette use in the US population.

**What is added by this report?**

We examined changes in menthol cigarette use among US adults who smoke. The prevalence of menthol cigarette use remains high for non-Hispanic Black adults who smoke and is increasing among other groups, especially Mexican American adults.

**What are the implications for public health practice?**

Increased menthol cigarette use among some demographic groups and ongoing prevalence disparities suggest that eliminating menthol cigarette use could strengthen ongoing health equity–related efforts to reduce US smoking prevalence.

## Introduction

Cigarette smoking is the leading cause of disease and death in the United States (1). Menthol flavor masks the unpleasant taste of tobacco and suppresses coughing impulses (2). Menthol in cigarettes increases tobacco use initiation and dependence in young people and reduces the likelihood for successful cessation (2–4). Restricting the sale of flavored tobacco products in the US has been shown to reduce the proportion of youth who try or use tobacco products (5–8). More than 18 million US adults smoked menthol cigarettes in 2019; it was estimated that prohibiting menthol cigarettes in the US would result in more than 1.3 million people quitting smoking, based on studies after menthol cigarettes were prohibited in Canada (9).

The commercial tobacco industry targets its marketing to certain populations. Neighborhoods with predominantly Black and lower-income residents have disproportionately higher numbers of advertisements and price promotions, in addition to the lowest pack prices for menthol cigarettes (10,11). Substantial differences have been noted in the prevalence of menthol cigarette use by sociodemographic group (12). Non-Hispanic Black adults, adults with lower income, and female adults have a higher prevalence of menthol cigarette use compared with people from other racial and ethnic groups, adults with higher incomes, and male adults, respectively (13,14). Furthermore, temporal changes in menthol cigarette use have varied across sociodemographic groups (15).

Monitoring menthol cigarette use can not only play a crucial role for program and regulatory planning but also inform the evaluation of programmatic and policy interventions for addressing disparities. By using 20 years of National Health and Nutrition Examination Survey (NHANES) data (1999–2018), we aimed to achieve 2 objectives: 1) investigate temporal and sociodemographic differences in the prevalence of menthol cigarette use and 2) examine the association of menthol cigarette use with self-reported health status among adults who smoke.

## Methods

### Participants

NHANES is an ongoing, cross-sectional, nationally representative survey of the civilian, noninstitutionalized US population, operated by the Centers for Disease Control and Prevention (16). It uses a stratified, multistage probability sampling design with oversampling of people aged 60 years or older, Black people, and Hispanic people to assess the health and nutritional status of adults and children in the United States. The data are collected continuously and released biennially. Participants are randomly selected for a home interview and then invited to participate in a medical examination at a mobile center. The overall response rates of the interviewed sample ranged from 52% to 84% during 1999–2018 (16). For this trend analysis, we excluded NHANES 2019–2020 data due to disruptions caused by the COVID-19 pandemic because these changes could bias the evaluation of temporal changes. NHANES data collection is approved by the Research Ethics Review Board of the National Center for Health Statistics (NCHS). All NHANES respondents provided consent before interview (16). The institutional review board’s approval was not required for this analysis of public data with de-identified individual records.

Tobacco use information during the home interview was collected only for adults 20 years or older, before 2013. We used 10 NHANES cycles from 1999 to 2018 and grouped them into 5 periods: 1999–2002, 2003–2006, 2007–2010, 2011–2014, and 2015–2018 (16). After excluding 72 (0.1%) participants who did not report smoking status, the final analytical sample included 11,431 participants who smoked cigarettes at the time of the survey (54.5% male, 45.5% female).

### Smoking status and menthol cigarette indicators

Interviewers collected cigarette smoking history and characteristics from adults during the home interview. Participants who ever smoked were defined as participants who answered yes to “Have you smoked at least 100 cigarettes in your entire life?”; otherwise, smoking status was defined as never smoked. Among participants who ever smoked, adults who currently smoke were defined as those who answered “every day” or “some days” to the follow-up question “Do you now smoke cigarettes?”; people were defined as having formerly smoked if they answered “not at all” to that question. In this study, we included only the participants who smoked “every day” or “some days” as adults who smoke. Participants who smoke were asked to show interviewers the pack of cigarettes they smoked. The cigarette brand was verified using the uniform product code (UPC) found on the cigarette pack presented. The UPC was matched to a database containing UPC and menthol designations. The current menthol cigarette use indicator (hereinafter, menthol cigarette use) was created with this matched information by NCHS (17).

### Covariates

Sociodemographic information collected during the interview included age, sex at birth (male and female), race and ethnicity, educational attainment, and family income-to-poverty ratio (IPR). We analyzed age in years as a 4-level variable: 20–29, 30–44, 45–64, and 65 years or older. Race and ethnicity had 5 groups: non-Hispanic White (hereinafter, White), non-Hispanic Black (hereinafter, Black), Mexican American, Other Hispanic American (hereinafter, Other Hispanic), and non-Hispanic Other races (hereinafter, Other).

Educational attainment and family IPR were used as indicators of participants’ socioeconomic position (SEP) throughout their lifetime (18). Educational attainment, a SEP indicator in young adulthood (19), was characterized as less than a high school graduate or high school graduate or higher. Family income at the time of interview, a current SEP indicator, was categorized according to the federal poverty thresholds of the US Department of Health and Human Services poverty guidelines (20). The family IPR was defined as the ratio of family income to the family’s appropriate poverty threshold at the time of interview and was divided into 3 categories: <1.3, 1.3 to <3.5, and ≥3.5. Self-reported health status was grouped into 3 categories: excellent, very good or good, and fair or poor. Health status was included as an indicator of a participant’s perception of overall health, which is related to SEP, lifestyle, and mortality (21,22).

### Statistical methods

By using menthol cigarette use as the response variable, binary logistic regression was used to estimate the crude and adjusted prevalence of menthol cigarette use among adults who smoke and compare between different subgroups by age, sex, race and ethnicity, educational attainment, health status, and survey period. The interaction terms of the survey period with other covariates were included in the model and used to estimate prevalence by period, racial and ethnic group, and other covariates (23). The prevalence ratio (PR) was estimated by using prevalence estimates. The middle year of each survey cycle was treated as a continuous variable to estimate average relative change and annual percentage change (APC) for temporal trend analysis. With year and year squared of survey cycle as continuous variables in logistic regression, the APC of prevalence was estimated by using the average annual marginal change (semi-elasticity) in prevalence from the logistic model.

Analyses accounting for the complex sampling design were conducted using Stata (version 17.0, StataCorp LLC). Interview sample weights were used to account for sampling design per NHANES analytic guidelines (16). We used multiple imputation (MI) with chained equations to impute the missing values of the menthol cigarette indicator (n = 699, 6.1% of all adults who smoked), IPR (n = 5,287, 9.6% of all adults), educational attainment (n = 92, 0.2% of all adults), and health status (n = 47, 0.1% of all adults). The imputation model of missingness included all the dependent and independent variables of logistic models plus sampling design variables (primary sampling unit, stratum). Twenty sets of multiple imputed data were generated to provide adequate reproducibility of MI analysis (24). The Stata MI module with survey data module of Stata was used for menthol cigarette use, IPR, education, or health status–related analyses. *P* values of 2-sided statistical tests <.05 or nonoverlapping 95% CIs suggested a significance for population inference and comparison across population subgroups.

## Results

During 1999–2018, 28.8% (95% CI, 27.2%–30.4%) of all US adults who smoked used menthol cigarettes annually (Table 1). Menthol cigarette use was higher among younger adults (aged 20–64 y), female adults, Black adults, and, on average, among adults with a lower IPR (Table 2). Among all adults who smoked cigarettes, the prevalence of menthol cigarette use increased significantly from 22.9% in 1999–2002 to 35.9% in 2015–2018 (APC = 3.8%; 95% CI, 2.7%–4.9%).

**Table 1:** Type of Cigarette Smoked Among US Adults Aged 20 Years or Older Who Smoke, by Sociodemographic Characteristics and Health Status, National Health and Nutrition Examination Survey, 1999–2018

US adults who smoke	All (N = 11,431)^a^	Nonmenthol cigarettes (n = 7,107)^a^	Menthol cigarettes (n = 3,625)^a^
**All**	100	71.2 (69.6–72.8)	28.8 (27.2–30.4)
**Age, y**			
20–29	23.8 (22.9–24.9)	22.5 (21.1–23.8)	28.6 (26.6–30.5)
30–44	33.7 (32.4–34.9)	33.8 (32.4–35.4)	33.6 (31.5–35.8)
45–64	35.2 (34.0–36.4)	36.0 (34.5–37.5)	32.2 (30.0–34.4)
≥65	7.3 (6.7–7.9)	7.7 (6.9–8.4)	5.6 (4.8–6.4)
**Sex**			
Male	54.5 (53.4–55.6)	57.1 (55.7–58.5)	45.9 (44.1–47.7)
Female	45.5 (44.4–46.6)	42.9 (41.5–44.3)	54.1 (52.3–55.9)
**Race and ethnicity**			
White, non-Hispanic	69.2 (66.9–71.6)	76.1 (73.7–78.4)	52.2 (48.9–55.6)
Black, non-Hispanic	13.1 (11.7–14.4)	5.2 (4.5–5.9)	32.9 (29.9–35.8)
Mexican American	6.7 (5.7–7.7)	7.7 (6.5–9.0)	4.1 (3.2–5.1)
Other, Hispanic	4.9 (3.8–6.1)	4.6 (3.3–6.0)	5.8 (4.4–7.1)
Other, non-Hispanic	6.1 (5.4–6.8)	6.4 (5.4–7.3)	5.0 (4.0–6.0)
**Educational attainment**			
Less than high school graduate	25.5 (24.3–26.8)	26.0 (24.4–27.5)	23.9 (22.2–25.6)
High school graduate or higher	74.5 (73.2–75.7)	74.0 (72.5–75.6)	76.1 (74.4–77.8)
Missing, n	15	8	6
**Income-to-poverty ratio**			
<1.30	33.3 (31.5–35.0)	30.8 (28.7–32.9)	37.2 (35.1–39.2)
1.30 to <3.5	38.4 (36.9–40.0)	38.9 (37.1–40.7)	38.3 (35.8–40.7)
≥3.5	28.3 (26.5–30.1)	30.3 (28.2–32.3)	24.5 (21.9–27.2)
Missing, n	1,042	633	357
**Health status**			
Excellent	11.7 (10.9–12.5)	11.4 (10.4–12.4)	12.3 (10.8–13.9)
Very good or good	64.3 (63.2–65.4)	65.2 (63.8–66.5)	63.3 (61.2–65.3)
Fair or poor	24.0 (22.9–25.1)	23.4 (22.1–24.8)	24.4 (22.6–26.2)
Missing, n	10	7	3

a Among 11,431 participants who smoke, 699 had missing data for menthol cigarette use. The percentages for all participants included all 11,431 participants, and other percentages by menthol cigarette status used 10,732 participants without missingness of menthol cigarette status. All values are weighted percentage (95% CI), unless otherwise indicated.

**Table 2:** Adjusted Prevalence^a^ of Menthol Cigarette Use Among US Adults Aged 20 Years or Older Who Smoke, by Sociodemographic Characteristics and Health Status, National Health and Nutrition Examination Survey, 1999–2018

US adults who smoke	All (N = 11,431)	1999–2002 (n = 2,170)	2003–2006 (n = 2,220)	2007–2010 (n = 2,665)	2011–2014 (n = 2,302)	2015–2018 (n = 2,074)	Annual percentage change^b^
**Menthol cigarette use, all**	28.6 (27.1–30.2)	22.9 (20.1–25.7)	27.1 (24.7–29.6)	28.0 (24.9–31.1)	30.8 (27.9–33.7)	35.9 (32.4–39.5)	3.8 (2.7 to 4.9)
**Age, y**							
20–29	35.6 (32.8–38.5)	25.5 (21.9–29.2)	31.1 (25.6–36.7)	36.1 (29.7–42.4)	42.7 (35.6–49.9)	44.3 (37.4–51.1)	5.1 (3.5 to 6.7)
30–44	28.7 (26.3–31.0)	23.8 (19.8–27.7)	26.5 (22.4–30.7)	25.9 (21.2–30.5)	27.9 (23.2–32.6)	40.3 (34.3–46.4)	4.3 (2.5 to 6.0)
45–64	25.6 (23.8–27.3)	21.2 (17.9–24.6)	25.3 (22.0–28.6)	26.1 (22.4–29.8)	27.1 (23.0–31.2)	28.4 (23.9–32.9)	2.8 (1.3 to 4.3)
≥65	22.1 (19.2–25.0)	18.3 (12.5–24.0)	25.9 (19.4–32.4)	20.5 (13.8–27.1)	22.1 (15.9–28.4)	23.8 (17.6–30.1)	1.7 (−1.0 to 4.5)
**Sex**							
Male	24.2 (22.6–25.8)	19.5 (16.7–22.2)	21.7 (19.1–24.3)	22.1 (18.9–25.2)	26.6 (23.2–30.0)	32.8 (28.9–36.8)	4.8 (3.3 to 6.2)
Female	34.0 (32.0–36.0)	27.0 (23.3–30.8)	33.6 (30.1–37.1)	35.0 (31.0–39.1)	35.8 (31.8–39.9)	39.6 (35.0–44.2)	2.6 (1.4 to 3.8)
**Race and ethnicity**							
White, non-Hispanic	21.5 (19.9–23.1)	14.0 (11.0–17.0)	19.8 (17.0–22.6)	22.4 (19.1–25.6)	23.4 (19.7–27.1)	29.3 (24.7–33.8)	4.2 (2.8 to 5.6)
Black, non-Hispanic	73.0 (70.9–75.2)	78.0 (73.5–82.5)	78.7 (74.0–83.4)	64.9 (59.4–70.4)	71.0 (65.9–76.0)	71.8 (67.7–75.9)	−0.8 (−1.3 to −0.3)
Mexican American	19.2 (16.2–22.3)	12.8 (7.3–18.2)	13.3 (6.7–20.0)	17.4 (11.4–23.3)	24.0 (16.0–32.0)	31.0 (23.5–38.5)	7.1 (4.0 to 10.3)
Other, Hispanic	32.8 (27.5–38.1)	23.6 (10.4–36.8)	22.7 (10.3–35.1)	35.0 (26.2–43.8)	42.2 (28.0–56.4)	43.0 (35.1–50.9)	4.3 (0.6 to 8.0)
Other, non-Hispanic	22.5 (18.4–26.6)	16.9 (7.2–26.7)	17.6 (10.0–25.3)	19.3 (10.9–27.7)	26.8 (17.0–36.7)	33.9 (24.0–43.7)	5.1 (1.5 to 8.8)
**Educational attainment^c^**							
Less than high school graduate	25.5 (23.3–27.7)	20.7 (17.2–24.2)	24.3 (20.1–28.4)	22.8 (18.2–27.3)	26.5 (21.6–31.5)	34.6 (28.6–40.5)	4.3 (2.5 to 6.1)
High school graduate or higher	29.8 (28.1–31.5)	23.7 (20.6–26.8)	28.1 (25.0–31.1)	29.7 (26.6–32.9)	32.2 (28.9–35.6)	36.4 (32.6–40.1)	3.6 (2.4 to 4.8)
**Income-to-poverty ratio^c^**							
<1.30	29.0 (27.0–30.9)	24.7 (20.3–29.1)	27.2 (23.9–30.6)	27.1 (23.2–31.0)	30.3 (26.8–33.8)	37.1 (32.7–41.6)	3.5 (2.0 to 5.0)
1.30 to <3.5	28.6 (26.6–30.6)	22.1 (18.8–25.4)	26.1 (22.6–29.6)	27.9 (23.8–32.1)	32.0 (27.6–36.4)	36.6 (32.0–41.2)	4.3 (2.9 to 5.7)
≥3.5	28.3 (25.5–31.0)	21.8 (17.4–26.2)	28.4 (23.8–33.1)	29.2 (23.2–35.1)	29.6 (22.9–36.4)	33.5 (25.0–42.0)	3.4 (1.3 to 5.4)
**Health status^c^**							
Excellent	29.2 (26.0–32.4)	26.1 (19.5–32.7)	32.1 (26.6–37.7)	27.2 (20.8–33.6)	29.8 (23.3–36.2)	31.7 (22.2–41.3)	1.5 (−1.0 to 4.1)
Very good or good	28.4 (26.7–30.1)	22.7 (19.7–25.7)	26.0 (23.4–28.6)	28.3 (25.1–31.5)	30.9 (27.8–34.0)	36.3 (31.5–41.0)	4.0 (2.7 to 5.3)
Fair or poor	28.6 (26.0–31.1)	21.8 (17.6–26.1)	27.8 (22.4–33.2)	27.6 (21.8–33.4)	30.8 (25.3–36.4)	37.0 (31.8–42.2)	4.3 (2.5 to 6.1)

a Adjusted prevalence ratio (PR) was estimated by using logistic regression with survey period, age group, sex, race and ethnicity, education, income-to-poverty ratio group, and health status group. 95% CIs that do not overlap between 2 PRs indicates significance. All values are percentage (95% CI), unless otherwise indicated.

b 95% CIs that do not overlap zero suggested a significant annual percentage change.

c Multiple imputed data sets were used for estimates related to education, income-to-poverty ratio, and health status.

The APC of menthol cigarette use among adults who smoke was significantly higher among younger adults, male adults, and adults with poorer health status. Adults aged 65 years or older had no significant change in the prevalence of menthol cigarette use. Black adults had a significant decrease in the prevalence of menthol cigarette use (APC = −0.8%; 95% CI, −1.3% to −0.3%). However, Black adults annually had the highest prevalence of menthol cigarette smoking among racial and ethnic groups. Other racial and ethnic groups, especially Mexican American adults, had a large increase in the prevalence of menthol cigarette use. Among Mexican American adults who smoked, the prevalence of menthol cigarette use increased from 12.8% (95% CI, 7.3%–18.2%) in 1999–2002 to 31.0% (95% CI, 23.5%–38.5%) in 2015–2018. The prevalence of menthol cigarette use increased across all levels of educational attainment and IPR. There was little temporal change in the prevalence of menthol cigarette use among adults who smoke who had excellent health status (APC = 1.5%; 95% CI, −1.0% to 4.1%). However, there was a significant temporal increase among adults with very good or good health status (APC = 4.0%; 95% CI, 2.7% to 5.3%) and with fair or poor health status (APC = 4.3%; 95% CI, 2.5%–6.1%) (Table 2).

The unadjusted prevalence of menthol cigarette use among adults who smoke increased by 60% from 1999–2002 to 2015–2018 (PR [2015–2018 vs 1999–2002] = 1.60; 95% CI, 1.30–1.90). Compared with adults aged 65 years or older, adults aged 20 to 29 years had a 51% higher prevalence of menthol cigarette use (PR = 1.51; 95% CI, 1.28–1.74) (Table 3). Compared with male adults, female adults had a 38% higher prevalence of menthol cigarette use during 1999 to 2018 (PR = 1.38; 95% CI, 1.29–1.47). Compared with White adults, Black adults had a 3.3 times higher prevalence of menthol cigarette use (PR = 3.31; 95% CI, 3.04–3.57). Adults with lower family income (IPR <1.30) had a 31% higher prevalence of menthol cigarette use than adults with higher family income (IPR ≥3.5) (PR = 1.31; 95% CI, 1.16–1.47). There was a lower prevalence of menthol cigarette use among adults with educational attainment of less than a high school diploma compared with those with a high school diploma or more and among adults with poorer health status compared with excellent health status.

**Table 3:** Adjusted Prevalence Ratio^a^ of Menthol Cigarette Use Among US Adults Aged 20 Years or Older Who Smoke, by Sociodemographic Characteristics and Health Status, National Health and Nutrition Examination Survey, 1999–2018

US adults who smoke (N = 11,431)	Unadjusted model	Age- and sex- adjusted model^b^	Age-, sex-, and race/ethnicity–adjusted model^c^	Fully adjusted model^d^
	Prevalence ratio (95% CI)			
**Period**				
1999–2002	1 [Reference]	1 [Reference]	1 [Reference]	1 [Reference]
2003–2006	1.16 (0.93–1.39)	1.18 (0.95–1.41)	1.20 (1.03–1.37)	1.18 (1.02–1.35)
2007–2010	1.26 (1.00–1.52)	1.26 (1.01–1.52)	1.22 (1.02–1.41)	1.22 (1.03–1.41)
2011–2014	1.37 (1.11–1.62)	1.40 (1.14–1.66)	1.36 (1.15–1.56)	1.34 (1.14–1.54)
2015–2018	1.60 (1.30–1.90)	1.66 (1.35–1.96)	1.59 (1.35–1.84)	1.57 (1.33–1.81)
**Age, y**				
20–29	1.51 (1.28–1.74)	1.61 (1.36–1.86)	1.67 (1.44–1.91)	1.61 (1.39–1.83)
30–44	1.28 (1.08–1.47)	1.33 (1.13–1.53)	1.34 (1.14–1.53)	1.30 (1.11–1.48)
45–64	1.18 (1.00–1.36)	1.21 (1.02–1.39)	1.19 (1.02–1.36)	1.16 (0.99–1.32)
≥ 65	1 [Reference]	1 [Reference]	1 [Reference]	1 [Reference]
**Sex**				
Male	1 [Reference]	1 [Reference]	1 [Reference]	1 [Reference]
Female	1.38 (1.29–1.47)	1.39 (1.30–1.48)	1.41 (1.32–1.49)	1.41 (1.32–1.49)
**Race and ethnicity^b^**				
White, non-Hispanic, %	1 [Reference]	1 [Reference]	1 [Reference]	1 [Reference]
Black, non-Hispanic, %	3.31 (3.04–3.57)	3.32 (3.07–3.58)	3.35 (3.09–3.61)	3.40 (3.14–3.66)
Mexican American, %	0.83 (0.67–0.99)	0.85 (0.70–0.99)	0.84 (0.70–0.98)	0.90 (0.74–1.05)
Other Hispanic, %	1.53 (1.22–1.84)	1.53 (1.26–1.81)	1.49 (1.24–1.74)	1.52 (1.27–1.78)
Other, non-Hispanic, %	1.11 (0.90–1.32)	1.08 (0.89–1.28)	1.05 (0.85–1.25)	1.05 (0.85–1.25)
**Educational attainment^b^**				
Less than high school graduate	1 [Reference]	1 [Reference]	1 [Reference]	1 [Reference]
High school graduate or higher	1.09 (1.00–1.17)	1.03 (0.95–1.11)	1.17 (1.08–1.26)	1.17 (1.07–1.27)
**IPR**				
<1.30	1.31 (1.16–1.47)	1.22 (1.07–1.36)	0.98 (0.88–1.08)	1.03 (0.91–1.14)
1.30 to <3.5	1.15 (1.01–1.29)	1.12 (0.98–1.25)	0.99 (0.88–1.10)	1.01 (0.90–1.12)
≥3.5	1 [Reference]	1 [Reference]	1 [Reference]	1 [Reference]
**Health status^a^**				
Excellent	1 [Reference]	1 [Reference]	1 [Reference]	1 [Reference]
Very good or good	0.93 (0.82–1.03)	0.89 (0.79–0.98)	0.94 (0.84–1.05)	0.97 (0.86–1.08)
Fair or poor	0.97 (0.84–1.10)	0.95 (0.83–1.07)	0.93 (0.82–1.05)	0.98 (0.85–1.11)

Abbreviation: IPR, income-to-poverty ratio.

a Multiple imputed data sets of IPR, education, and health status were used.

b Adjusted prevalence ratio (PR) was estimated by using logistic regression with variables (age group, sex, and survey period) and interaction terms of these variables with survey period.

c Adjusted PR was estimated by using logistic regression with all variables (age group, sex, race and ethnicity, and survey period) and interaction terms of these variables with race and ethnicity and survey period.

d Adjusted PR was estimated by using logistic regression with all variables (age group, sex, race and ethnicity, educational attainment, income-to-poverty ratio group, health status, and survey period) and interaction terms of these variables with race and ethnicity and survey period.

The PRs changed little after adjusting for age group and sex (Table 3). However, after additional adjustment for race and ethnicity, the PR of adults who were high school graduates or higher (vs less than high school graduate) increased from 1.03 (95% CI, 0.95–1.11) to 1.17 (95% CI, 1.08–1.26), and the PRs of menthol cigarette use among different income groups were no longer significant (IPR <1.30 vs IPR ≥3.5, PR = 0.98; 95% CI, 0.88–1.08). Female adults who smoke continued to have a significantly higher prevalence of menthol cigarette use than male adults who smoke (PR = 1.41, 95% CI, 1.32–1.49).

## Discussion

Among US adults who smoke, the prevalence of menthol cigarette use increased from 22.9% in 1999–2002 to 35.9% in 2015–2018. Mexican American adults had the highest increase in menthol cigarette use during this period. Although the prevalence of menthol cigarette use declined among Black adults (from 78.0% to 71.8%), menthol cigarette use in this group remained substantially higher than in any other racial and ethnic group. Female adults and adults with a high school diploma or more were more likely to use menthol cigarettes than male adults and adults with less education than a high school diploma, respectively.

The commercial tobacco industry markets menthol cigarettes to specific population groups, including young people, women, and racial and ethnic minority groups, with a particular focus on Black communities; these strategies involve the use of advertisements, giveaways, lower pricing, lifestyle branding, and event sponsorships (25). Notably, menthol cigarettes are more commonly found and are cheaper in neighborhoods with higher proportions of Black residents, younger people, and low-income households (11). These marketing efforts have likely contributed to menthol cigarettes being smoked disproportionately by certain population groups, such as adolescents, Black adults, and female adults (26). In addition, the menthol flavor increases the likelihood of youth and young adults experimenting with smoking, compared with the appeal of nonmenthol cigarettes (27). People who smoke menthol cigarettes are also less likely to successfully quit smoking (2,28). These challenges may be even more pronounced among Black people who smoke, who have a higher prevalence of menthol cigarette use compared with other population groups (2).

In contrast to the national population, a larger proportion of Black people are protected by any local policies that prohibit the sale of flavored tobacco products; however, a smaller proportion of Black people are protected by flavored tobacco policies that specifically prohibit the sale of menthol cigarettes (29). Based on policy outcome evaluation studies and other analyses, prohibiting the sale of menthol cigarettes in the United States would reduce cigarette smoking overall, including among Black people (7,9,30). However, given other social determinants of cigarette smoking and anticipated industry shifts to adjust to a new marketplace, it will be important to monitor the effects of a menthol cigarette sales prohibition on the smoking behavior of all population groups — including Black adults, given the history of marketing and given their higher prevalence of smoking menthol cigarettes (10,11).

This study shows that Black adults had the highest prevalence of menthol cigarette use throughout the study period, compared with other racial and ethnic groups, which is consistent with previous studies (15). The prevalence of menthol cigarette use increased significantly among other racial and ethnic groups, particularly among Mexican American adults, and among younger adults and persons who reported fair to poor health status. Multiple factors may correspond to these temporal changes, such as changes in the commercial tobacco industry’s marketing strategy and its targeted populations. In 1999, a large part of the US cigarette industry’s advertising and promotional expenditures were for activities such as favorable stocking of products in retail stores, or offering “buy one, get one” incentives to receive cigarette or noncigarette products, while in 2021 most expenditures were for retailer price discounts to reduce cigarette prices for consumers (31). These changes can disproportionately affect certain population groups, given that tobacco retailers are clustered in lower income neighborhoods and in neighborhoods with a high proportion of youth and racial and ethnic minority groups (32). It is worth noting that when people who smoke menthol cigarettes seek medical attention, increased clinical opportunities exist to promote and provide comprehensive, barrier-free tobacco cessation services.

A previous study showed that adults with lower income have a higher risk of starting and continuing to use menthol cigarettes (15). Our results showed that lower income was associated with a higher prevalence of menthol cigarette use, though this relationship was attenuated after additional adjustments for race and ethnicity. These findings align with recent research, which suggests that once a person has established a dependence on smoking, the continued preference for menthol cigarettes is more strongly associated with subjective personal satisfaction and reward, rather than income level (33). These findings suggest that race and ethnicity could have a stronger association with menthol cigarette use than income.

This observational study has several limitations. First, we used multiyear, nationally representative, cross-sectional survey data that cannot establish causality. Second, sociodemographic characteristics and smoking behavior were self-reported and may be subject to bias. Third, current SEP was represented using IPR. IPR accounts for inflation over time; however, it did not account for other factors such as standard of living, taxes, and variation among geographic locations, which affect SEP over time (34). In addition, 10% of the participants did not report their income. We imputed missing data by assuming that the information was missing at random, which might not be sufficient to eliminate the potential information bias. Because of the significant disruption caused by the COVID-19 pandemic, we chose not to include NHANES 2019–2020 data in this temporal trend report. Finally, we did not have sufficient information to estimate menthol cigarette use among some groups with high prevalence of tobacco use, including Native American and Alaska Native people; Asian, Native Hawaiian, or Pacific Islander people; and LGBTQ+ people. Additional studies are needed to assess menthol cigarette use among a broader range of sociodemographic groups and among people who may belong to more than one population group.

We conclude that, from 1999 to 2018, Black adults had the highest prevalence of menthol cigarette use. Additionally, we found a notable increase in the use of menthol cigarettes among adults who smoke — particularly Mexican American adults, younger adults, and those who reported fair to poor health status. These subgroups may be at heightened risk of use of menthol cigarettes. Implementing policies that prohibit the sale of menthol cigarettes, alongside promoting and ensuring access to comprehensive and barrier-free tobacco cessation services, can reduce cigarette smoking, including among population groups experiencing tobacco use disparities. Notably, more jurisdictions have prohibited the sale of menthol tobacco products following the period covered in our study (35). Continued monitoring of menthol cigarette use is important to track progress in advancing health equity in the United States.
